# Nerve Ultrasound for the Diagnosis of Tarsal Tunnel Syndrome: Findings in 26 Clinically and Electrophysiologically Confirmed Feet

**DOI:** 10.3390/jcm15051699

**Published:** 2026-02-24

**Authors:** Ben-Ole Holtz, Mihai Ceanga, Andrea Behnert, Raphaela Marquardt, Christian Geis, Hubertus Axer

**Affiliations:** Department of Neurology, Jena University Hospital, Friedrich Schiller University, 07747 Jena, Germany; mihai.ceanga@med.uni-jena.de (M.C.); andrea.behnert@med.uni-jena.de (A.B.); raphaela.marquardt@med.uni-jena.de (R.M.); christian.geis@med.uni-jena.de (C.G.); hubertus.axer@med.uni-jena.de (H.A.)

**Keywords:** tarsal tunnel syndrome, nerve ultrasound, nerve conduction studies

## Abstract

**Background/Objectives**: Posterior tarsal tunnel syndrome is a compressive neuropathy of the tibial nerve at the level of the ankle within the tarsal tunnel. However, there is no established gold standard for the diagnosis of tarsal tunnel syndrome to date. High-resolution ultrasound could add important value in this setting. But up to date, to the best of our knowledge, only six clinical studies have investigated the use of ultrasound for the diagnosis of tarsal tunnel syndrome, with partially conflicting results. Most authors identify nerve swelling at the level of anatomical compression as the key ultrasonographic criterion, whereas at least one study and some expert opinions instead emphasize nerve compression at the site of entrapment. **Methods**: We performed a retrospective observational study of high-resolution ultrasound of the tibial nerve in patients with typical clinical and electrophysiological characteristics of tarsal tunnel syndrome. **Results**: A cohort of 26 feet with clinically and electrophysiologically confirmed tarsal tunnel syndrome was collected. Nerve ultrasound demonstrated a moderate sensitivity of 65% for the detection of abnormalities of the tibial nerve when applying the commonly used cut-off of 11.8 mm^2^ for the tibial nerve at the level of the tarsal tunnel entry or within the tarsal tunnel. In all but one of the cases classified as pathological on ultrasound, an increase in tibial nerve CSA in the tarsal tunnel was observed compared with the CSA measured 5–10 cm proximal to the tarsal tunnel entry (by a factor of 1.6 ± 0.53). A secondary cause was found in only 12% of the cases. But this study also suggests that ultrasound may remain unremarkable in approximately one third of patients with tarsal tunnel syndrome. **Conclusions**: Establishing the diagnosis of tarsal tunnel syndrome remains challenging. Our study supports the hypothesis proposed in previous publications that tarsal tunnel syndrome appears to be an exception among compression neuropathies on ultrasound: sonography demonstrates nerve swelling not proximal to the site of compression, but at the level of the anatomical compression. Further prospective data would be of substantial clinical relevance.

## 1. Introduction

Posterior tarsal tunnel syndrome is a compressive neuropathy of the tibial nerve at the level of the ankle within the tarsal tunnel [1]. Clinically, patients report burning dysesthesias or numbness confined to the plantar aspect of the foot [2]. Symptoms are typically exacerbated either by mechanical load (prolonged standing or walking) or by rest, presumably due to reduced venous return [3]. Accordingly, tarsal tunnel syndrome represents an important differential diagnosis to polyneuropathy, which usually presents in a stocking-like distribution, and to plantar fasciitis [4].

Robust epidemiological data are currently lacking. Overall, tarsal tunnel syndrome is considered rare and appears to be strongly dependent on the presence of specific risk factors. For example, a Dutch study published in 2018 reported a surprisingly high prevalence of just over 40% among patients with diabetes [5,6]. However, this high prevalence in diabetic subjects may be driven by the existence of diabetes-related neuropathy as a concomitant risk factor. Additional risk factors described include obesity and frequent jogging [6,7]. In view of the heterogeneous epidemiological data, some authors have suggested that the true prevalence may be underestimated.

From an etiological perspective, current evidence suggests that a secondary cause can be identified in approximately 60–80% of cases [8]. In particular, intra- and extraneural ganglion cysts; an accessory flexor digitorum longus muscle or an accessory soleus muscle; osteophytes; varicosities; trauma; and foot deformities have been repeatedly reported [9,10,11,12]. The remaining 20–40% of cases are therefore classified as idiopathic tarsal tunnel syndrome.

**Figure 1:**
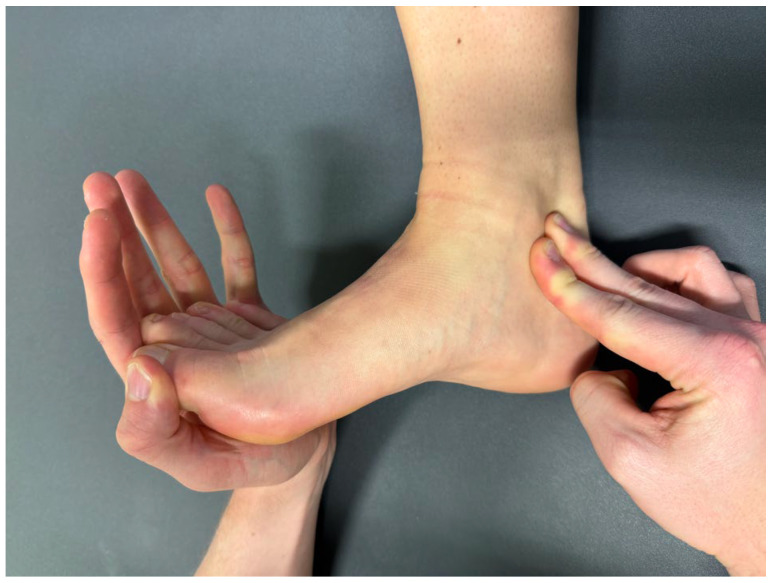
The dorsiflexion–eversion test is used during the physical examination. The ankle is everted and dorsiflexed as much as possible. All metatarsophalangeal joints are maximally dorsiflexed. The test is considered positive if it provokes pain or numbness in the distribution of the tibial nerve.

Upon clinical examination, a positive Hoffmann–Tinel sign over the tarsal tunnel can be elicited, with a reported sensitivity of 50–60% and a specificity of 80–90% [6,13]. However, its diagnostic value appears to be limited in the presence of concomitant polyneuropathy [14]. Symptoms should be reproducible or exacerbated by a nerve compression test over the tarsal tunnel [2]. In particular, a positive dorsiflexion–eversion test (Figure 1) has been described as having very high sensitivity and specificity [15]. However, this specific test has not yet been routinely used in our neurological outpatient clinic and ultrasound service.

Electrophysiological testing has notable limitations in the diagnostic workup. Motor nerve conduction studies of the tibial nerve are recommended, in particular, with recordings from the abductor hallucis brevis muscle and, if necessary, additionally from the abductor digiti quinti muscle. Sensory nerve conduction studies of the medial and lateral plantar nerves can also be performed, with stimulation proximal to the tarsal tunnel syndrome (TTS) and recording from the first or fifth digit. The sensitivity of nerve conduction studies is reported to be approximately 50–82%, with a specificity of 80–90% [16,17,18]; fractionated nerve conduction studies, with nerve conduction velocity measurements restricted to the segment across the tarsal tunnel, may also be applied [18], and, in our experience, have proven to be the most sensitive neurophysiological technique. Electromyography is not routinely used [17].

Magnetic resonance imaging (MRI) is highly specific for identifying secondary causes. However, its sensitivity for detecting pure nerve compression is lower, as functional alterations are often not visible [11]. In addition, nerve ultrasound is increasingly being used routinely in clinical practice.

From a therapeutic perspective, conservative measures such as orthoses, nonsteroidal anti-inflammatory drugs, physiotherapy, and, if required, ultrasound-guided perineural injections with local anesthetics are employed [2]. In the absence of clinical improvement or in the presence of neurological deficits, surgical decompression of the tibial nerve and all its branches by division of the flexor retinaculum, with additional removal of space-occupying lesions or secondary causes if present, is indicated [19,20].

Overall, there is no established gold standard for the diagnosis of tarsal tunnel syndrome; in particular, idiopathic tarsal tunnel syndrome remains a diagnostic challenge. In our neuromuscular ultrasound laboratory, however, we regularly evaluate patients with suspected tarsal tunnel syndrome in whom the clinical diagnosis appears quite plausible. In these cases, electrophysiological testing often demonstrates reduced nerve conduction velocity precisely across the tarsal tunnel.

By contrast, the findings on ultrasound examination are subjectively often less striking. In particular, the literature on precise ultrasound diagnostic criteria for tarsal tunnel syndrome is limited and partly contradictory: some reviews and expert opinions suggest that tarsal tunnel syndrome appears on ultrasound analogous to carpal tunnel syndrome [21]: with nerve swelling proximal and distal to the tarsal tunnel and an hourglass-shaped compression within the tunnel. To date, however, this pattern has not been consistently confirmed in clinical studies. The aim of this study was therefore to conduct an in-depth review of the literature to identify valid ultrasound diagnostic criteria for tarsal tunnel syndrome and, subsequently, to compare these findings with the data collected in our cohort to date.

## 2. Materials and Methods

### 2.1. Retrospective Observational Study

We performed a retrospective analysis of ultrasound data from the past ten years (2016–2025) in our institution (Department of Neurology, Jena University Hospital). The study was approved by the local ethics committee (ethics committee of the Friedrich-Schiller-University Jena, number 2025-4011-BO-D).

Patients who had been referred to ultrasound evaluation with suspected tarsal tunnel syndrome were identified. Subsequently, the corresponding discharge letters and electrophysiological findings were reviewed to first determine whether the clinical criteria were fulfilled.

The clinical criteria comprised numbness or dysesthesias confined exclusively to the plantar aspect of the foot with sparing of the dorsum of the foot and without clinical evidence of a polyneuropathic syndrome. Second, it was assessed whether the electrophysiological criteria were also met. In addition, nerve conduction studies must not reveal any evidence of polyneuropathy (exclusion criterium).

Only patients who fulfilled both the clinical and electrophysiological criteria for tarsal tunnel syndrome were included. In addition, polyneuropathy had to be explicitly excluded, either on neurological examination or by nerve conduction studies.

Overall, 48 patients with suspected tarsal tunnel syndrome were referred to our nerve ultrasound clinic between 2016 and 2025. Of these, five patients did not undergo electrophysiological testing and were therefore excluded. Four patients were excluded due to insufficiently documented or non-compatible neurological examination findings. In 22 patients, the electrophysiological criteria for tarsal tunnel syndrome were not met, or the nerve conduction studies additionally revealed evidence of polyneuropathy.

Thus, 26 affected feet with clinically and electrophysiologically confirmed tarsal tunnel syndrome from 17 patients were identified using this approach. As the dorsiflexion–eversion test was not consistently documented in these cases, this clinical test was not analyzed in this study.

### 2.2. Measurements

**Figure 2:**
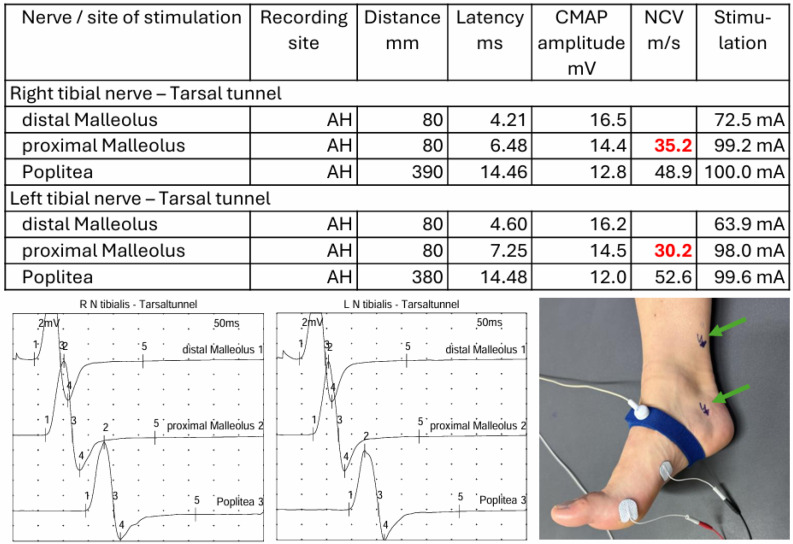
Example of nerve conduction studies in a patient with bilateral tarsal tunnel syndrome. Note that nerve conduction velocity was determined in a fractionated manner, both across the tarsal tunnel segment (see image at bottom right corner with stimulation sites (arrows) proximally and distally to the tarsal tunnel) and along the remaining course of the nerve. Red numbers indicate decreased nerve conduction velocity.

Nerve conduction studies were recorded using a Natus Nicolet EDX^®^ EMG-/ENG-/EP-/IOM-System, and analysis was performed using Natus Elite^®^ Electrodiagnostics Software (Synergy 15.0; Viasysy Healthcare, Natus Europe GmbH, Planegg, Germany). Electrophysiological workup of tarsal tunnel syndrome involves motor nerve conduction studies of the tibial nerve with recording from the abductor hallucis brevis muscle, including fractionated stimulation across the tarsal tunnel. Stimulation is performed distal to the tarsal tunnel and proximal to the tarsal tunnel; the distal stimulation site is located 8 cm from the recording electrode over the abductor hallucis brevis muscle, and the proximal stimulation site is placed a further 8 cm proximal to the first stimulation site. An additional motor stimulation is performed at the popliteal fossa. Subsequently, the motor nerve conduction velocity across the tarsal tunnel is compared with the proximal nerve conduction velocity and interpreted with reference to internal normative values or by side-to-side comparison. The cut-off of normal motor nerve conduction velocity of the tibial nerve is 40.6 m/s, and the nerve conduction velocity over the tarsal tunnel has to be at least 10 m/s slower than over the shank. Delayed nerve conduction velocity over the tarsal tunnel is considered indicative of tarsal tunnel syndrome (Figure 2).

Nerve ultrasound was performed by experienced neurologists on a Toshiba/Canon Aplio i800 Machine with a PLT-1204BT linear array transducer (14–18 MHz, Canon Medical Systems, Otawara, Tochigi, Japan). Nerve cross-sectional measurements were performed with the embedded software on Toshiba/Canon Aplio i800. Cross-sectional area (CSA) of the tibial nerve was measured 5–10 cm prior to the tarsal tunnel (TT), at the entry of the TT and in the TT. The location of the maximal compression was evaluated, and the echogenicity of the nerve was evaluated throughout the cases. Changes observed in the longitudinal view, particularly abrupt changes in caliber and loss of echogenicity, were considered supportive criteria for the identification of neuropathy. Other structural features, such as changes in fascicular organization, were not systematically documented and were, therefore, not analyzed in this retrospective study.

### 2.3. Statistics

All data were analyzed with the Statistical Package for the Social Sciences software (SPSS version 31.0; IBM Deutschland GmbH; Ehningen, Germany). The values were presented as mean and standard deviation (SD), and numbers and percentages. First, we described the cohort using descriptive statistics.

Spearman correlations were used to analyze correlations between nerve conduction studies and peripheral nerve ultrasound. For all analyses, a *p*-value < 0.05 was considered statistically significant.

## 3. Results

In total, 17 patients (10 women and seven men) were included for the period from 2016 to 2025. The median age of the patients was 60 years. Approximately 50% of cases involved bilateral disease (9/17 patients); overall, exactly 13 right and 13 left feet were examined.

Clinically, in accordance with the inclusion criteria, all patients reported numbness or dysesthesias confined exclusively to the plantar aspect of the foot. In seven patients (10 feet, 38%), symptoms worsened with physical load, in two patients (four feet, 15%) at rest, and in two patients (three feet, 12%) both at rest and with physical load (Table 1). A Hoffmann–Tinel sign over the tarsal tunnel was documented in four patients; in the remaining cases, it was not documented whether the sign was not assessed or whether it was absent.

**Figure 3:**
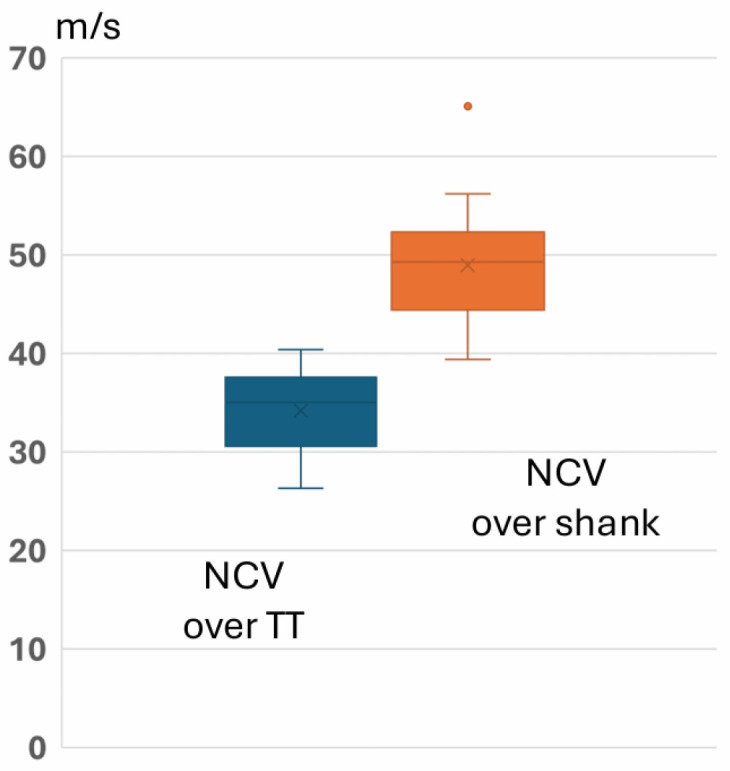
Box plots of nerve conduction velocity (NCV) over the tarsal tunnel (TT) and over the shank. The orange dot refers to one outlier with high NCV.

Nerve conduction velocity over the tarsal tunnel (34.2 ± 4.1 m/s) was significantly lower than over the shank (49.0 ± 5.7 m/s, paired *t*-Test: T = −8.267, *p* < 0.001; see Figure 3).

There are large normative studies addressing the normal cross-sectional area of the tibial nerve at the level of the ankle. In 2023, Senarai et al. conducted a systematic review and, averaging across 48 studies, reported an upper CSA limit of 11.8 mm^2^ for the tibial nerve at the ankle in healthy adults [22]. Using this cut-off, ultrasound demonstrated an increased CSA at the tarsal tunnel entry or within the tarsal tunnel itself in 16 of 26 feet (58%, Table 2). In addition, a CSA cut-off value of 15 mm^2^ was reached in only 27%, and a cut-off value of 19 mm^2^ was reached in only 8% of our cases.

The point of maximal nerve enlargement was located at the tarsal tunnel entry in 56% of cases (nine feet) and within the tarsal tunnel in 44% (seven feet). Propagation of nerve swelling into the plantar nerves was observed in six feet (38%), involving the medial plantar nerve in five cases and the lateral plantar nerve in one case.

A secondary cause was identified in 12% of cases. In two cases, a large compressive or displacing artery was observed, and in one case, a constricting connective tissue structure was identified. In one of these cases, no absolute CSA increase was present; thus, identification of a secondary cause was used as the primary ultrasound criterion. However, in this specific case, a relative increase in CSA compared with the segment proximal to the tarsal tunnel was observed (9.9 vs. 8.5 mm^2^), further supporting the ultrasound diagnosis.

**Figure 4:**
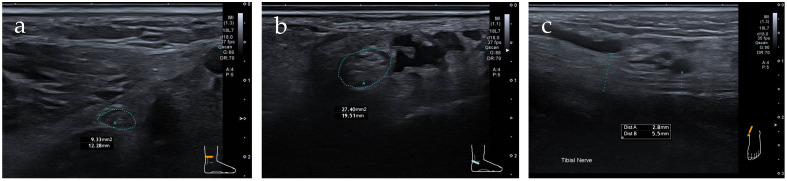
Nerve ultrasound of the tarsal tunnel. (**a**) Cross-sectional view of the tibial nerve approximately 7 cm proximal to the tarsal tunnel entry (blue circle). (**b**) Cross-sectional view of the tibial nerve in the proximal portion of the tarsal tunnel (blue circle). Particular attention should be paid to the significant increase in cross-sectional area (CSA) from 9.3 mm^2^ (**a**) to 27.4 mm^2^ (**b**). (**c**) Corresponding longitudinal view of the tibial nerve (right = proximal; left = distal), demonstrating marked caliber enlargement (blue lines) and loss of the fascicular architecture as a correlate of nerve injury.

Among nerves classified as pathological, the mean CSA at the level of the tarsal tunnel or at the tarsal tunnel entry was 15.9 mm^2^ (±3.9 mm^2^). Longitudinal caliber compression was observed in six feet, while findings were equivocal in two additional feet (31% in total). A clear reduction in echogenicity was seen in 15% of cases. On average, a 1.6 ± 0.53-fold increase in maximal CSA within the tarsal tunnel was observed compared with the segment 5–10 cm proximal. A decrease in CSA of the tibial nerve at the tarsal tunnel entry or within the tarsal tunnel was observed in only one of the cases classified as pathological. The CSA increase and caliber enlargement are illustrated exemplarily in Figure 4.

In theory, a relevant side-to-side difference in CSA could also serve as a diagnostic criterion. However, in our cohort, there was not a single case in which a relevant side-to-side CSA difference was present without the absolute threshold values also being exceeded.

Accordingly, the overall sensitivity of ultrasound was 65%. Accordingly, ultrasound failed to detect any abnormality (CSA enlargement, secondary cause change in echogenicity) in 35% of cases. In 100% of these sonographically unremarkable cases, the CSA of the nerve proximal to the tarsal tunnel was greater than that within the tarsal tunnel. This underscores that comparing the CSA with a more proximal reference value provides relevant diagnostic value.

**Figure 5:**
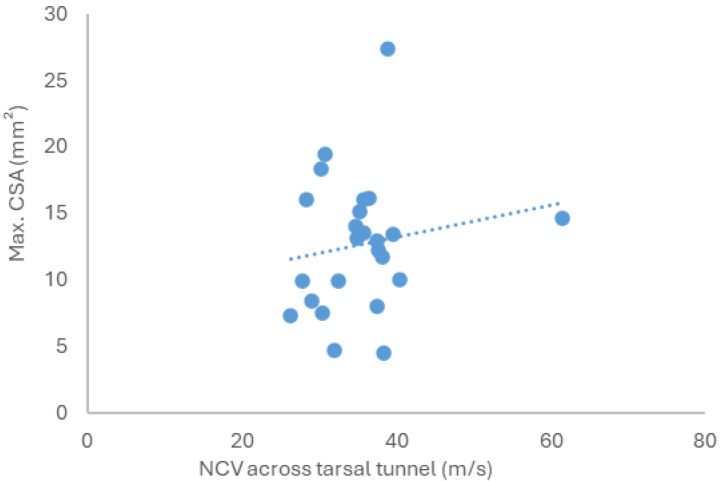
Correlation of maximum cross-sectional area (CSA) of the tibial nerve in tarsal tunnel or at tarsal tunnel entry with nerve conduction velocity (NCV) across tarsal tunnel. Each data point represents paired values of CSA and NCV for a single tibial nerve of one foot.

Seven of the twenty-six feet were additionally examined by MRI, and none demonstrated pathological findings. Delay of tibial nerve conduction velocity across the tarsal tunnel did not significantly correlate with the degree of nerve swelling (Spearman’s ρ = 0.126, *p* = 0.279, Figure 5).

## 4. Discussion

### 4.1. Review of the Literature

Table 3 summarizes nerve ultrasound findings in clinical case series of patients with tarsal tunnel syndrome.

The first study to systematically describe ultrasound findings in tarsal tunnel syndrome was published by Nagaoka in 2005 [23]. In this study, 17 feet from 17 patients who had undergone ultrasound examination between 1988 and 2003 were analyzed. All cases showed at least pathological electrophysiological findings, most commonly in the form of abnormal sensory nerve conduction studies to the hallux. On ultrasound, a secondary cause was identified in 100% of cases (13 ganglion cysts, three cases of varicosities, and one talocalcaneal coalition). As a limitation, it should be noted that the data are very old. At that time, it was not yet common practice to assess the cross-sectional area (CSA) of a nerve. Moreover, examinations were performed using a 10 MHz transducer (whereas current examinations are typically conducted with probes of at least 18 MHz). Consequently, the results are of limited usefulness for establishing diagnostic criteria and primarily underscore the importance of nerve ultrasound for the detection of secondary causes.

The largest cohort of patients with tarsal tunnel syndrome was reported by Fantino in 2014 [25], comprising 81 patients. However, the sole inclusion criterion in this study was clinically diagnosed tarsal tunnel syndrome. Only 25 of these patients underwent electrophysiological testing, of whom only 13 showed pathological findings (52% of those tested, corresponding to 16% of all included patients). In addition, a major limitation is that the focus of this study was again clearly on the identification of secondary causes, which were detected in 69 of 81 cases (85%). Of the remaining 12 cases, seven were described as having a “neuropathy,” characterized by a hypoechoic increase in CSA within the tarsal tunnel, while five cases showed no abnormalities. However, precise CSA values, either as absolute or relative cut-off thresholds, were not provided in this study.

The study by El Shazly published in 2011 [24] differs from the others in some respect. Only cases with clinically and electrophysiologically confirmed tarsal tunnel syndrome were included. However, in contrast to all other studies, the authors reported a marked compression of the nerve within the tarsal tunnel on ultrasound (3.31 mm^2^ vs. 8.02 mm^2^ in healthy controls). No comparison with a more proximal nerve segment was performed, and only idiopathic tarsal tunnel syndromes were considered.

In 2016, Samarawickrama et al. [26] likewise exclusively investigated patients with clinically and electrophysiologically confirmed tarsal tunnel syndrome. Over a nine-year period, six patients with nine affected feet were identified. The mean CSA of the tibial nerve within the tarsal tunnel was increased, with an average of 16.1 mm^2^. Tibial nerve swelling was observed in 89% of cases. In three of these cases, the swelling extended into the lateral plantar nerve, and in one case, into the medial plantar nerve. In one case, swelling was confined to the plantar nerves alone. No comparison with the nerve segment proximal to the tarsal tunnel was performed. A secondary cause was identified in 67% of the feet, with swelling in some cases extending into the medial plantar nerve (33%) and the lateral plantar nerve (44%).

In 2016, Tawfik et al. [27] compared 14 patients with tarsal tunnel syndrome to 17 healthy controls and demonstrated a significant enlargement of the tibial nerve within the tarsal tunnel. A diagnostic cut-off value of 19 mm^2^ for the CSA within the tarsal tunnel was proposed, yielding a sensitivity of 61%. In addition, a ratio between the CSA proximal to the tarsal tunnel and the CSA within the tarsal tunnel was calculated, with a cut-off value of >1 considered pathological (i.e., whenever the nerve was not compressed at this site), resulting in a sensitivity of 74%.

The most recent study on this topic was again published by Fantino in 2021 [28]. This time, the focus explicitly included the cross-sectional area (CSA) of the nerve. Fantino described 23 patients with 27 affected feet, which were compared with 21 healthy controls. Only clinically and electrophysiologically confirmed cases were included. The CSA within the tarsal tunnel was markedly increased in these patients, measuring 20.1 ± 8.8 mm^2^ compared with 10.3 ± 2.3 mm^2^ in healthy controls. A cut-off value of 15 mm^2^ was proposed in combination with an increase in CSA of +5 mm^2^ compared with the nerve segment 10 cm proximal to the tarsal tunnel. In agreement with the ratio described by Tawfik, healthy controls also showed, on average, a slight decrease in CSA when comparing the segment proximal to the tarsal tunnel with the segment within the tarsal tunnel (−0.2 mm^2^). In particular, a CSA increase of 5 mm^2^ was highlighted as yielding a sensitivity of 81% and a specificity of 100%.

### 4.2. The Retrospective Study

In our experience, not all patients benefit from surgical treatment of a presumed tarsal tunnel syndrome; therefore, particular importance should be attributed to accurate diagnosis. Our study demonstrates that nerve ultrasound, even in clinically and electrophysiologically confirmed cases in which polyneuropathy has additionally been excluded, typically reveals only mild abnormalities. In approximately one third of cases, sonography remained without definite pathological findings.

Assuming that the diagnostic gold standard is electrophysiology, the sensitivity of nerve ultrasound to detect a tarsal tunnel syndrome is smaller. Deceleration of nerve conduction velocity as a correlate of focal demyelination may be detectable earlier than nerve swelling in the tarsal tunnel. In addition, the compression of the tibial nerve in the tarsal tunnel (as well as consecutive nerve swelling) may be changing (and not permanent) due to the individual amount of physical stress and activity but may lead to earlier local demyelination of the nerve.

We further demonstrate that the available evidence for ultrasonographic diagnostic criteria and typical findings in tarsal tunnel syndrome are very limited. To date, only six clinical studies have systematically described nerve ultrasound in tarsal tunnel syndrome. Of these, Samarawickrama et al. evaluated only six patients [27]; Nagaoka et al. collected their data predominantly in the 1980s and 1990s, when parameters such as nerve CSA were not yet routinely established [23]; and El Shazly et al. reported a decrease in tibial nerve CSA within the tarsal tunnel [24], yielding results entirely divergent from those of the remaining studies. The largest study, including 81 patients, conducted by Fantino, was able to electrophysiologically confirm tarsal tunnel syndrome in only 16% of cases [25] and likewise did not systematically assess nerve CSA or caliber.

Consequently, the studies by Tawfik et al. [27] and Fantino [28] remain particularly relevant. Both consistently describe swelling of the tibial nerve at the level of the tarsal tunnel. Our data are in agreement with these findings. However, the proposed CSA cut-off values of 15 mm^2^ and 19 mm^2^ were reached by only 27% and 8% of our patients. These cut-offs therefore appear less suitable than the threshold of 11.8 mm^2^ proposed by Senarai et al. based on the evaluation of 48 studies, which yielded a sensitivity of at least 58% in our cohort. However, this cut-off refers to neuropathy in general rather than specifically to tarsal tunnel syndrome.

The proposed relative criteria were met with highly variable frequency. Thus, only 19% of patients fulfilled the criterion proposed by Fantino et al. [28] of a CSA increase of at least 5 mm^2^. The ratio of >1 described by Tawfik [27] was met by 46% of patients. For both relative criteria, it should be noted that a CSA measurement proximal to the tarsal tunnel was not obtained in every case. When correcting for the fact that a proximal CSA measurement was available in only 21/26 cases, the resulting sensitivities were 10% (CSA difference > 5 mm^2^) and 57% (ratio > 1), respectively.

The frequency of secondary causes identified in our cohort was markedly lower than that reported in the literature (12% compared with 52–100%, Table 1). This proportion could not be increased even after retrospective re-evaluation of the ultrasound images, arguing against inadequate detection of secondary causes by the examiners. As nerve ultrasound at our institution is still an adjunctive examination and is not performed routinely in all patients, it can be speculated that predominantly unclear or mild cases were referred to ultrasound evaluation. Cases showing more pronounced abnormalities either clinically or on MRI may have proceeded directly to surgery without prior nerve ultrasound. For instance, patients subjected directly to surgical correction of adult-acquired flatfoot, which is one of the mechanical deformities most frequently associated with compression of the tibial nerve in the tarsal tunnel [29], may not have been examined by nerve ultrasound.

In some cases, compression of the tibial nerve within the tarsal tunnel was described in our reports (longitudinal view, 31%). However, upon retrospective review of these images, this finding was not always unequivocally reproducible. In particular, as the tibial nerve frequently divides into its terminal branches already within the tarsal tunnel, a longitudinal view at this level can easily create the impression of caliber compression. If, however, no nerve swelling distal to this presumed constriction can be demonstrated, the finding appears questionable. At a minimum, in such cases, it should be demonstrated that the apparent compression is located clearly proximal to the point of nerve bifurcation. The other studies do not propose a clear solution to this issue.

MRI data of seven feet only were available in this study, and so a reliable comparison between nerve ultrasound and MRI could not be done based on our data. Measurements of nerve CSA are not routinely done in MRI data, and MRI of the foot may emphasize changes in bones, joints, and connective tissue, therefore giving it additional diagnostic value in the evaluation of tarsal tunnel anatomy.

In our view, whenever a visual impression of caliber compression is present, the spatial relationship between the site of apparent compression and the branching point of the tibial nerve should at least be reported. The pathophysiological explanation of the observation that maximal CSA increase is most often located at the level of the tarsal tunnel or its entry remains speculative. Mechanisms such as local mechanical constraints, venous congestion, or dynamic changes in fascicular arrangement within the tunnel may contribute [30]. It would be of interest for future studies to investigate how well the visual impression of caliber compression correlates with surgical outcome after decompression, and how reliably the absence of caliber compression on nerve ultrasound predicts a lack of therapeutic benefit from decompression. If a secondary cause such as a ganglion cyst or a similar lesion is identified, the finding of caliber compression would likely be of lesser relevance, and treatment of the secondary cause should take priority.

### 4.3. Limitations

The retrospective nature and the relatively small sample size are clear limitations of this study. The number of examined feet was relatively small (n = 26); however, when considering only clinically and electrophysiologically confirmed cases, our cohort represents the second-largest published study to date. This is attributable to the rarity of tarsal tunnel syndrome and to the difficulty of limited sensitivity of nerve conduction studies. Moreover, the analysis was retrospective, resulting in heterogeneity of the ultrasound parameters assessed; in some cases, the division into the medial and lateral plantar nerves was measured, whereas in others, it was not. In total, six different examiners were involved, further reducing the uniformity of the collected data. The frequent lack of visualization of the plantar nerves is particularly noteworthy, as Samarawickrama et al. [26] reported that in 11% of cases, nerve swelling was confined exclusively to the plantar nerves without involvement of the tibial nerve. In addition, in some cases, the CSA of the nerve 5–10 cm proximal to the tarsal tunnel was not determined, precluding the application of relative diagnostic criteria. It remains speculative whether the overall sensitivity of nerve ultrasound would have been higher if all of these parameters had been consistently assessed in every case. In this study, the fascicular structure of the tibial nerve was not systematically analyzed. However, high-resolution ultrasound does not necessarily depict individual fascicles one-to-one, and larger hypoechoic structures may represent the merging or clustering of multiple fascicles rather than true single fascicles. This has been demonstrated in studies comparing HRUS with histology and magnetic resonance microscopy [31].

## 5. Conclusions

In summary, we identified 26 feet with clinically and electrophysiologically confirmed tarsal tunnel syndrome, representing the second-largest cohort published to date. In this cohort, nerve ultrasound demonstrated a moderate sensitivity of 65% for the detection of tarsal tunnel syndrome when applying the commonly used cut-off of 11.8 mm^2^ for the tibial nerve at the level of the tarsal tunnel entry or within the tarsal tunnel. In all but one case classified as pathological on ultrasound, an increase in tibial nerve CSA was observed compared with the CSA measured 5–10 cm proximal to the tarsal tunnel entry. A secondary cause was identified in 12% of cases.

To date, only six clinical studies have described ultrasound criteria for the diagnosis of tarsal tunnel syndrome. Five of these six studies consistently report swelling of the tibial nerve at the level of the tarsal tunnel. This observation is noteworthy, as the ultrasound pattern appears to resemble that of a peripheral nerve lesion rather than a classic compressive neuropathy. An hourglass-shaped nerve compression, which would be expected in a compressive neuropathy, has been described in case reports but not in the clinical studies analyzed. Instead, the typical finding seems to be nerve swelling, frequently associated with damaging secondary causes. In such cases, the condition might also be described as a tibial neuropathy at the level of the tarsal tunnel. At present, it remains speculative whether the absence of an hourglass sign truly predicts the absence of relevant compression; if so, surgical decompression by division of the retinaculum would be unlikely to be effective.

In conclusion, establishing the diagnosis of tarsal tunnel syndrome remains challenging, particularly in mild-to-moderate cases. Our study supports the hypothesis proposed in previous publications that tarsal tunnel syndrome appears to be an exception among compression neuropathies on ultrasound: sonography demonstrates nerve swelling not proximal to the site of compression, but at the level of the anatomical compression. Further prospective data—especially regarding the correlation between ultrasound findings, intraoperative findings, and postoperative outcomes—would be of substantial clinical relevance.

## Figures and Tables

**Table 1:** Demographic and clinical characteristics of the study population.

Baseline Characteristics	
Patients/feet (n)	17/26
Age (mean/standard deviation)	60 ± 13.5
Male/female (%)	41%/59%
Right feet/left feet (n)	13/13
Plantar numbness or paresthesia	100%
Increasing symptoms …	
- at exertion	38%
- at rest	15%
- at exertion and rest	12%

**Table 2:** Summary of key ultrasonographic criteria for the diagnosis of tarsal tunnel syndrome.

No. of Patients/Feet	CSA 5–10 cm Prior TT (mm^2^)	CSA at TT Entry (mm^2^)	CSA in TT (mm^2^)	Location of Max. CSA	Compression (Caliber)	Echogenicity at TT Entry or in TT	Secondary Cause	No Abnormal Findings
1/1		14.6	11	Entry	Yes	Normal		
1/2	8.5		9.9	In TT	No	Normal	Yes	
2/3	9.2	11.8	13.5	In TT	Questionable	Normal		
3/4	8.5	13.1	10.8	Entry	Questionable	Normal		
4/5	10.7		10.6	Prior TT	No	Normal		X
4/6	10.8		9.9	Prior TT	No	Normal		X
5/7	10.1	12.3	16	In TT	Yes	Normal		
5/8	8.9	12.9		Entry	Yes	Normal		
6/9		14		Entry	No	Normal		
7/10	11.7	15.1	6.8	Entry	Yes	Reduced	Yes	
7/11	10.4	18.3	16.5	Entry	Yes	Reduced		
8/12		16.1	10.3	Entry	-	Normal		
9/13	9.3	27.4	20.4	Entry	No	Normal	Yes	
10/14	7		16	In TT	Yes	Normal		
11/15	10	9.5	11.9	In TT	No	Normal		
11/16	11.4	12.4	19.4	In TT	No	Reduced		
12/17	10.6	13.4		Entry	-	Normal		
13/18	13.6	10	9.9	Prior TT	No	Normal		X
13/19	12.6	9.2	12.2	In TT	No	Normal		
14/20	10.1	7.5		Prior TT	No	Normal		X
14/21	8.3	7.3		Prior TT	No	Normal		X
15/22	7.8	4.7		Prior TT	-	Normal		X
15/23		4.5		Entry	-	Normal		X
16/24		15.2	17.2	In TT	-	Hypoechoic		
17/25	9.8	8		Prior TT	No	Normal		X
17/26	9.2	8.4		Prior TT	No	Normal		X
Average all	9.9 ± 1.6	12.8 ± 5.0						
Average pathological	9.9 ± 1.5	15.3 ± 3.9						
Pathological results (%)		62%			31%	15%	12%	35%

Abbreviations: CSA, cross-sectional area; TT, tarsal tunnel.

**Table 3:** Summary of relevant studies on the use of nerve ultrasound for the diagnosis of tarsal tunnel syndrome.

Reference	Patients/Feet, n	Controls, n	Electrophysiologically Confirmed	Secondary Causes Found	Mean-CSA mm^2^ at TT Pat. vs. Control	Mean-CSA mm^2^ 5–10 cm Prior TT Pat. vs. Control	Swelling in TT Described	Overall Ultrasound Sensitivity
Nagaoka, 2005 [23]	17/17	-	100%	100%	-	-	-	100%
El Shazly, 2011 [24]	10/11	14/28	100%	-	3.31/8.02	-	No	-
Fantino, 2014 [25]	81/81	-	16%	85%	-	-	Yes	94%
Samarawickrama, 2016 [26]	6/9	-	100%	67%	16.1/-	-	Yes	100%
Tafwik, 2016 [27]	23/14	17/17	100%	-	20.6/13.8	16.8/15.6	Yes	74–83%
Fantino, 2021 [28]	23/27	21	100%	52%	20.1/10.3	10.4/10.6	Yes	81%

Abbreviations: CSA, cross-sectional area; TT, tarsal tunnel.

## Data Availability

The data used to support the findings of this study are available from the corresponding author upon request.

## Abbreviations

The following abbreviations are used in this manuscript:

CMAP	Compound motor action potential
CSA	Cros-sectional area
NCS	Nerve conduction study
NCV	Nerve conduction velocity
SD	Standard deviation
TT	Tarsal tunnel
TTS	Tarsal tunnel syndrome
